# Marital Conflict, Family Socioeconomic Status, and Depressive Symptoms in Migrant Children: A Moderating Mediational Model

**DOI:** 10.3390/bs13060441

**Published:** 2023-05-24

**Authors:** Liuhua Ying, Yanli Wang, Shasha Yu

**Affiliations:** 1 Department of Psychology, Zhejiang Sci-Tech University, Hangzhou 310018, China; 2 Nantong Zhangjian No.1 Primary School, Nantong 226000, China

**Keywords:** marital conflict, family SES, communication, peer attachment, depression, migrant children

## Abstract

The present study examines the roles of parent–child communication and peer attachment in the relationships between marital conflict, family socioeconomic status (SES), and depressive symptoms in migrant children. The present study was a cross-sectional design. A total of 437 children were selected from 2 public schools of migrant children, and they were assessed on measures of marital conflict, family SES, parent–child communication, peer attachment, and depressive symptoms. Results showed that peer attachment moderates the relationships between marital conflict, parent–child communication, and depressive symptoms. That is, for migrant children with high peer attachment, marital conflict influences depressive symptoms directly, but also indirectly through parent–child communication. For migrant children with low peer attachment, marital conflict only exerts a direct influence on depressive symptoms. In addition, parent–child communication mediates the relationship between family SES and depressive symptoms, although the mediating effects were not significant for groups with a high or a low level of peer attachment. Thus, parent–child communication serves as one critical pathway, linking marital conflict, or family SES, with depressive symptoms. Furthermore, peer attachment acts as a buffer against the negative effects of marital conflict on depressive symptoms.

## 1. Introduction

The psychological adaptation of migrant children is always an important research topic. In China, there is a large number of migrant children aged 0–18 years who leave their hometowns with their parents, for host cities [1,2,3,4]. These migrant children are more likely to suffer from psychological maladjustment in new cities. Depression is one of the most common internalized symptoms in migrant children in China [5,6,7], although other studies show no more severe depressive symptoms in migrant children than in their urban counterparts. For example, previous studies have shown that migrant children might experience higher levels of psychological, social, and emotional difficulties (i.e., depression, anxiety, aggression, and suicide) than their non-migrant urban counterparts [7,8]. Guo and colleagues (2015) reported that the rate of depression was 20.1% in a sample of 568 migrant children from 8 to 12 years of age [9].

Family socioeconomic status (SES) is usually a multidimensional construct which describes their level of access to, and control over, social and economic resources such as education, occupation, and income [10]. Family SES is considered as a risk factor for depressive symptoms. Children with low family SES are more likely to be exposed to major stressors and negative life events out of their control, and have access to less psychological support, thus increasing the risk of depression [11]. To date, there is a large number of studies which demonstrate that low SES was related to a higher risk of depression [12]. For example, Cao and colleagues found that low family SES was related to greater depressive symptoms in adolescents [13].

Marital conflict is characterized by disagreements, arguments, and disputes between parents, and is the key element in determining child adjustment [14]. Children’s exposure to destructive marital conflict is directly related to internalizing problems, both during childhood and later in life [15,16,17,18]. Within Chinese culture, family characteristics may be especially influential on children’s development, particularly where adversity is involved [3]. However, during the adjustment to new and unfamiliar environments, migration increases the potential risk of suffering from a variety of stresses, and thus, of migrant parents experiencing more interpersonal conflicts. Family tension has been found to be a risk factor for psychological issues in migrant children [19].

On the other hand, the “spillover” hypothesis proposes that marital quality affects (spillover) parent–child relationship quality [20]. To date, some researchers suggest that marital conflict influences children’s psychological adjustment indirectly by altering parenting practices and the quality of the parent–child relationship [20,21,22]. Nonetheless, little is known about the role of parent–child communication in the path from marital conflict to adolescents’ development. Parent–child communication is often defined as those verbal or nonverbal communications which occur between parents and children within a family system [23]. A communication pattern between parents and children is proposed to be influenced by marital conflicts and family SES. For example, Grych (2002) showed that parents in a relationship marked by conflict, coped with this stress by often reducing the frequency of communication and providing more negative parenting interactions [24]. Additionally, low family SES was also found to increase financial stress and leave parents with less time for, and frequency of, communication with their children [25]. Less frequent or ineffective communication between parents and children was found to increase the risk of negative psychological and behavioral outcomes for children [26,27,28,29,30]. For example, parent–child communication played a mediated role in the relationship between the marital quality of parents and school adjustment in Korean adolescents [31]. Thus, it seems reasonable to hypothesize that marital conflict or family SES might influence depressive symptoms in migrant children through parent–child communication.

Attachment was originally referred to as a lasting affective bond established between a child and their primary caregiver [32,33]. However, with their emerging autonomy from family during later childhood and adolescence, adolescents begin to rely on peers as sources of companionship and emotional support [34,35,36,37]. Stronger attachment to peers has been found to promote adolescents’ psychological adjustment, including higher self-esteem, greater life satisfaction, and fewer emotional and behavioral problems [35,38,39,40].

Furthermore, as peers play an increasingly important role in children’s adjustment, peer attachment is more likely to shape the nature of the family life, and moderate children’s experiences in the family setting. To date, fewer studies have investigated protective factors of peer attachment against the development of depression. For example, a previous study showed that peer attachment might serve as a buffer against the adverse effects of caregiver psychological distress on youth depression, as higher attachment to peers was significantly correlated with lower levels of depression [41]. Similarly, another study (2009) found that those adolescents who were suffering from interparent conflict within the family, but also had better connections with peers in school, had a higher level of positive affect than their counterparts [42]. On the other hand, according to the “compensation” hypothesis, peer attachment may act as a buffer, for the adverse effect of low-frequency and ineffective communication between parent and child, on adolescents’ psychopathology, although fewer studies have investigated the protective role of peer attachment in the mediating path. For example, Zhang et al. (2017) showed that positive peer attachment may compensate for a problematic relationship with parents through the establishment of supportive relationships with peers instead [43].

Therefore, the present study examines the roles of parent–child communication and peer attachment in the relationships between marital conflict, family SES, and depressive symptoms in migrant children. Specifically, we hypothesize that (1) parent–child communication mediates the relationship between marital conflict or SES and depressive symptoms; and (2) the direct and indirect (as mediated by parent–child communication) paths from marital conflict or SES to depressive symptoms were moderated by different levels of peer attachment.

## 2. Methods

### 2.1. Participants and Procedure

The participants of the current study comprised of 437 students (240 boys and 197 girls) attending their 4th or 5th grade (with a mean age of 10.87 years old, *SD* = 0.72). Of these participants, the majority of the children (93%) came from non-divorced families with biological, nonadoptive children. Only 12% (*n* = 51) of the fathers and 8.5% (*n* = 37) of the mothers had a college degree or higher. In addition, most families had a monthly income of more than 2280 RMB, the official low-income threshold per month for a family, whereas only a total of 34 families (7.8%) had a monthly income of less than 2000 RMB.

The participants were selected from the two public schools for migrant children in two labor-importing cities (i.e., Hangzhou and Jiaxing) in May 2016 (see Ying et al., 2023 for full details) [44]. In China, migrant schools in some cities enroll these children with lack of urban household registration status (hukou). These schools are usually considered to have inferior facilities and less well-equipped faculties [45]. After participants and their parents gave their written informed consent, a total of 444 participants sat in the classroom and took over half an hour to complete a series of anonymous questionnaires, including the Children’s Perceptions of Interparental Conflict Scale (CPICS) [46,47], the Parent–Child Communication Questionnaire (PCCQ) [48], the Inventory of Parent and Peer Attachment (IPPA) [49], and the Center for Epidemiologic Studies Depression Scale for Children (CES-DC) [50]. The investigator provided participants some explanations for items with which they had difficulty understanding. A total of 437 participants completed all measures, yielding a response rate of close to 98.4%. This study was approved by the local education authorities (i.e., County Departments of Education), school principals, and the Research Ethics Committee of the research institute.

### 2.2. Measures

#### 2.2.1. Family SES

Paternal and maternal education levels were coded into five categories: (a) primary school, (b) junior high school, (c) high school, (d) a bachelor’s degree or higher. Family income was coded into 6 categories: (a) Below 1000 RMB, (b) 1000 RMB–2000 RMB, (c) 2000 RMB–4000 RMB, (d) 4000 RMB–6000 RMB, and (e) above 6000 RMB. In addition, according to the standard established by Shi and Shen (2007), paternal and maternal occupation were, respectively, coded into five categories: (a) casual laborer, unemployed, non-technical and agricultural laborers, (b) physical labor workers, self-employed staff, technical workers, or other similar workers, (c) clerical workers, shop assistants, skilled manual workers, and so on, (d) middle managers or professional and technical personnel, such as teachers, doctors, and technicians, and (e) government officials, senior management personnel, professional and technical personnel. In the current study, 87 (19.9%) participants did not report their household income. Thus, only paternal and maternal education levels and occupations were standardized and averaged to estimate a total measure of family SES.

#### 2.2.2. Marital Conflict

A Chinese version of the CPICS was used to assess the frequency and intensity of overt conflict that occurs between their parents when children are present. As for children who live in single-parent families, they were asked to report their perceptions of parents’ conflicts when their parents had lived together. This scale consisted of 10 items. Participants responded to each item on a 5-point Likert scale, ranging from 1 (never) to 5 (always), with high scores indicating greater levels interparental conflict. Previous studies have shown that the CPICS has a good degree of reliability and psychometric properties [46,47]. In the present study, the Cronbach’s alpha coefficient of the whole scale was 0.89.

#### 2.2.3. Parent–Child Communication

The 23-item PCCQ was used to assess 4 aspects (i.e., open expression, listening to parents, conflict resolution, and mutual understanding) of parent–child communication [48]. Sample items included “When I am depressed or upset, I will tell my parents”, and “I can handle conflicts with my parents well”. Participants responded to each item on a 5-point Likert scale, ranging from 1 (not at all) to 5 (very well), with higher scores indicating greater frequency and intensity of communication between parent and child. Previous studies have shown that the internal consistency and construct validity of this questionnaire were good [26,48]. In the present study, the Cronbach’s alpha coefficient of the entire scale was 0.94.

#### 2.2.4. Peer Attachment

The 25-item IPPA was designed to evaluate peer attachment based on 3 dimensions: trust, communication, and alienation [49]. Participants responded to each item on a 5-point Likert scale, ranging from 1 (almost never) to 5 (almost always), with higher scores indicating a higher quality of relationship with peers. Previous studies have shown that the original [49,51] and the Chinese version [6] of the IPPA had satisfactory psychometric properties. In the present study, the Cronbach’s alpha coefficient of the entire scale was 0.91.

#### 2.2.5. Depression

The Chinese version [8,50] of the CES-DC [52] was used to assess the children’s depressive symptoms during the previous week. Participants responded to each item on a 4-point Likert scale, ranging from 0 (not at all) to 3 (a lot), with higher scores indicating higher levels of depressive symptoms. Previous studies have found that both the original and the Chinese version of the CES-DC had satisfactory psychometric properties [8,50,52]. For the current sample, the Cronbach’s alpha coefficient of the entire scale was 0.89.

### 2.3. Data Analysis

Firstly, the means, standard deviations, and correlation coefficients of these research variables were calculated. Then, to examine the mediating effect of parent–child communication in the relationship between marital conflict or family SES and depressive symptoms, we conducted structural equation modeling analyses with the AMOS 22.0 software using the maximum likelihood (ML) iteration procedure [53]. When these values of the Tucker–Lewis index (TLI) [54], the comparative fit index (CFI) [55], and the incremental fit index (IFI; Bollen, 1989) exceeded 0.95 [56] and the root mean square error of approximation (RMSEA) was less than 0.06 [57], the model fit with the data was viewed as being good. Then, after obtaining the best fitting model, the bootstrap-biased correction, with 5000 samples which could be performed in the bootstrap estimation procedure in AMOS 17.0 software, was calculated to test the estimated mediating effect [58,59,60]. The mediating effects occurred when the CI did not contain zero [61].

Finally, to further examine whether peer attachment moderated the indirect path (i.e., marital conflict or family SES-parent–child communication-depressive symptoms), we examined group invariance in the final path model. Specifically, an unconstrained model, in which the path coefficients were allowed to vary across low (below the mean on peer attachment) and high (above the mean on peer attachment) peer attachment groups, was compared by using the Chi-square difference test with a constrained model in which these path coefficients were constrained to be equal.

## 3. Results

Weston and Gore (2006) suggested that data are considered normal if skewedness is less than 3 and kurtosis is less than 10 [62]. Data of the present study met the criteria. The means, standard deviations, and correlation coefficients of these research variables were presented in Table 1. Marital conflict was positively correlated with depressive symptoms (*r* = 0.35) but negatively correlated with peer attachment (*r* = −0.28), and the four aspects (i.e., open expression, listening to parents, conflict resolution, and mutual understanding) of parent–child communication (*r*’s = −0.28 to −0.49). The four aspects of parent–child communication were positively correlated with peer attachment (*r*’s = 0.46 to 0.48) but negatively correlated with depressive symptoms (*r*’s = −0.40 to −0.43). Family SES was significantly and positively correlated with peer attachment (*r* = 0.18) but not significantly correlated with other main study variables. In addition, the participants’ age was negatively correlated with the open expression of communication (*r* = −0.11), but positively correlated with depressive symptoms (*r* = 0.17). However, gender was not significantly correlated with the main study variables.

Next, structural equation modeling was performed to examine whether parent–child communications mediated the relationships between marital conflict, SES, and depressive symptoms. First, following Holmbeck’s (1997) suggestions [63], we tested the direct effect of marital conflict or SES on depressive symptoms and found that both marital conflict and SES were significantly related to children’s depressive symptoms. Then, we developed the fully saturated model, in which the mediator of parent–child communication was added. Specifically, in this fully saturated model, while SES and marital conflict served as two observed and exogenous variables, parent–child communication (a mediator) and depressive symptoms (an outcome variable) served as two latent and outcome variables, which were measured by their respective indicators. In addition, age has also been added into this fully saturated model as a covariate variable. The results showed that the model fitted the data well (χ^2^ (37) = 101.07, χ^2^/*df* = 2.732, *p* < 0.001, CFI = 0.971, TLI = 0.957, IFI = 0.971, RMSEA = 0.063). Specifically, marital conflict was significantly and directly related to depressive symptoms and indirectly related via parent–child communication. Additionally, SES was not significantly and directly related to depressive symptoms but indirectly related via parent–child communication. The bias-corrected bootstrapping analysis showed that the indirect path from marital conflict to depressive symptoms, via parent–child communication, was significant because the 95% CI did not contain zero (coefficient = 0.60, 95% CI = [0.34, 0.94], *p* < 0.001). Similarly, the indirect path from SES to depressive symptoms, via parent–child communication, was significant because the 95% CI did not contain zero (coefficient = −0.15, CI = [−0.32, −0.02], *p* < 0.05).

Finally, to further examine whether peer attachment moderated the indirect effect, we examined group invariance in the full model, across the low and high levels of peer attachment. First, considering that higher scores indicated a good quality of peer attachment, we specified the group with high peer attachment (*n* = 228; above the mean) and the group with low peer attachment (*n* = 209; below the mean). Then, in the unconstrained model, these path coefficients were allowed to vary across the high and low levels of peer attachment. In the constrained model, these path coefficients were constrained to be equal across the two groups. The Chi-square difference test showed that the fit of the constrained model (χ^2^ (87) = 154.74, χ^2^/*df* = 1.779, *p* < 0.001, CFI = 0.963, TLI = 0.954, IFI = 0.964, RMSEA = 0.042) was significantly poorer than that of the unconstrained model (χ^2^ (74) = 125.40, χ^2^/*df* = 1.695, *p* < 0.001, CFI = 0.972, TLI = 0.959, IFI = 0.973, RMSEA = 0.040; Δχ^2^ (13) = 29.34, *p* < 0.01).

**Figure 1:**
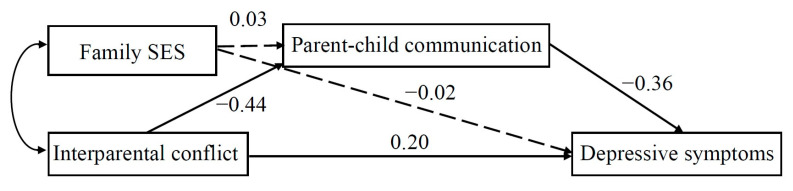
The path-analytic model depicting relations among interparental conflict, family SES, parent–child communication, and depressive symptoms in children with a high level of peer attachment. Note. The full arrow is the significant path; the dashed arrows are the nonsignificant paths. The values were standardized path coefficients.

**Figure 2:**
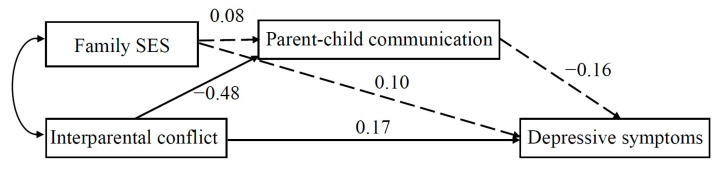
The path-analytic model depicting relations among interparental conflict, family SES, parent–child communication, and depressive symptoms in children with a low level of peer attachment. Note. The full arrow is the significant path; the dashed arrows are the nonsignificant paths. The values were standardized path coefficients.

As shown in Figure 1 and Figure 2, for migrant children with low peer attachment, only the effect of marital conflict on depressive symptoms, the indirect paths from marital conflict or SES on depressive symptoms—through parent–child communication— were not significant. However, for migrant children with high peer attachment, while the direct path from marital conflict to depressive symptoms was significant, the effect of marital conflict on depressive symptoms, through parent–child communication, was also significant. Further analysis, using the bias-corrected bootstrapping method, showed that the indirect effect from marital conflict to depressive symptoms, via parent–child communication among migrant children with high peer attachment, was significant because the 95% CI did not contain zero (coefficient = 0.58, 95% CI = [0.15, 1.17]).

## 4. Discussion

The current study examined the roles of parent–child communication and peer attachment in the relationships between marital conflict or family SES and depressive symptoms in migrant children. First, consistent with these previous studies [15,16,17], the results of the present study show that marital conflict is bivariately related to children’s depressive symptoms, suggesting that there is a deleterious effect of marital conflict on depressive symptoms. Furthermore, even after the inclusion of mediators (i.e., parent–child communication), the negative effect of marital conflict on depressive symptoms remained significant. Thus, these findings suggest that exposure to destructive conflict in the interparental relationship will increase the possibility of developing depressive symptoms in migrant children.

Additionally, consistent with the research hypotheses, marital conflict was found to partially relate to depressive symptoms, through parent–child communication, in migrant children. This finding offers more supports for the “spillover” hypothesis in migrant children in China. That is, the distress and frustration from marital conflict, occurring between parents, will spill over into parenting and the parent–child communication, thus leading to psychological maladjustment in children [20,64]. Communication in the parent–child relationship is related to children’s adjustment. For example, positive communication, such as talking frequently and being open, listening, and being responsive, is related to a good quality of the parent–child relationship, high self-esteem, and positive psychological outcomes [26,27,28,30]. In a migrant family, the conflict between parents, during the adjustment to a new and unfamiliar environment, might decrease the quality and frequency of parent–child communication by reducing parents’ sensitivity to the needs of their children and eliciting more negative responses from parents [24,65,66]. In turn, this can lead to a greater risk of depressive symptoms being experienced by migrant children. Thus, communication in the parent–child relationship is an important mediator in the path from marital conflict to positive psychological adjustments in migrant children.

Furthermore, the indirect path from marital conflict to depressive symptoms, via peer attachment, was found to vary across the different levels of peer attachment. That is, for migrant children with high peer attachment, marital conflict influences children’s depressive symptoms directly or indirectly, through parent–child communication. There is only a direct effect of marital conflict on depressive symptoms in migrant children with low peer attachment. This completely confirmed the research hypotheses on the buffering effect of peer attachment, suggesting that peer attachment might serve as a protective factor to alleviate the negative effects of marital conflict on depressive symptoms. According to attachment theory, children resort to peers as a source of emotional support and as a secure base in the transition from later childhood to adolescence [37,67]. Thus, security in the attachment to peers and feelings of support in close relationships are beneficial to the psychological health of adolescents [36]. However, after entering cities, migrant children need to rebuild close peer relationships to meet these developmental demands, such as gaining attention and emotional support. Social rejection by local peers, and the movement of parents’ worksite from one site to another, harm their relations with peers and foster a lack of belonging [3,68]. As a result, when exposed to marital conflict, migrant children with high peer attachment are more likely to maintain open and effective communication with their parents, which, in turn, alleviates the severity of depressive symptoms. On the contrary, the finding of the only direct effect of martial conflict on depressive symptoms suggests that migrant children with low peer attachment have greater vulnerability in the face of interparent conflicts, and are more likely to report depressive symptoms.

In addition, consistent with a previous study [69], the results of the current study indicated that SES affected depressive symptoms through parent–child communication, suggesting that parent–child communication plays a similar mediating role in the relationship between family SES and depressive symptoms. According to the conservation of resources theory [70], the low level of family SES increases financial stress, leaves parents with less time, and leads to a reduced frequency of communication with their children, all of which increases migrant children’s susceptibility to depressive symptoms. However, the mediating effect of parent–child communication, in such cases, is reduced within both groups with a high and a low level of peer attachment. This finding suggests that, in spite of children with high or low peer attachment, marital conflict—instead of family SES—exerts a greater influence on depressive symptoms, through parent–child communication.

Finally, consistent with a previous study [71], the results of the present study show that age is significantly and positively correlated to depressive symptoms. This finding suggests that depressive symptoms gradually become more prevalent during the transition from childhood to early adolescence. Thus, it might be necessary to devise age-appropriate interventions for depressive symptoms in migrant children. In addition, consistent with previous studies [72,73], this finding shows that age is negatively and significantly correlated to the open expression of parent–child communication. One possible explanation is that, as children increasingly develop individuality, they tend to keep secrets from their parents, in order to meet their need for autonomy, leading to a reduction in the frequency and intensity of open expression. In addition, the results of the present study show that maternal educational level is significantly and positively related to peer attachment and mutual understanding within parent–child communication. It is because maternal education shapes a set of proximal and distal parenting practices (e.g., high warmth and acceptance), which, in turn, increases mutual understanding between parent and child, as well as the possibility of building an affective bond to peers [74].

### 4.1. Limitations of the Study

Several limitations were worthy of be note. The first limitation was that all variables were assessed using self-reporting measures, which might bolster the relationships among these constructs, due to a common reporting method variance. Second, marital conflict was reported by adolescents but not by their parents; a previous study indicated that adolescents might tend to report a higher level of marital conflict than their parents [75]. Additionally, the data of the present study came from a convenience sample, which did not allow the research to control possible confounding variables, such as ethnicity, class, and school. Other important variables, such as perceived discrimination, were not included in the current study. Thus, generalizing the findings should be performed with caution. Finally, the correlational nature of our data did not allow us to draw a causal inference regarding the relationships between marital conflict and depressive symptoms.

### 4.2. Implications for the Practice

The findings of the present study provide some important information for school psychologists, or psychological service providers, to alleviate the depressive symptoms of migrant children in China. Specifically, the findings of the present study suggest that, for migrant children living in families experiencing high levels of conflict, parents might utilize clear, direct, and responsive communication to promote their children’s psychological adjustment. Thus, improving parents’ communication skills might be an appropriate target for alleviating the internalizing symptoms among migrant children [76]. For example, parents were trained to react promptly and sensitively to a child’s needs, feelings, and interests, and develop positive communication skills (e.g., listening and being open). Similarly, in the community, psychological service providers should provide some courses for migrant parents to improve the quality of their communication with children. In addition, of the array of potential resources, schools are a suitable location for interventions. As for those migrant children experiencing low peer attachment, school-based interventions, such as peer tutoring, were also used to alleviate the detrimental effect of marital conflict on depressive symptoms, by the development of supportive and accepting relationships with peers [77].

## 5. Conclusions

In summary, the findings of the current study have shown that marital conflict, instead of family SES, exerts more influence on depressive symptoms, through parent–child communication. Furthermore, peer attachment moderated the relationships between marital conflict, parent–child communication, and depressive symptoms in migrant children. That is, for migrant children with high peer attachment, marital conflict directly influences children’s depressive symptoms, but also indirectly influences them through parent–child communication. For migrant children with low peer attachment, marital conflict exerts a direct influence on depressive symptoms.

## Figures and Tables

**Table 1:** Descriptive statistics and intercorrelations among main variables.

Variables	Mean	*SD*	1	2	3	4	5	6	7	8	9	10
1. Age	10.87	0.72	-									
2. Gender	-	-	0.01	-								
3. Family SES	0.00	0.62	0.07	0.02	-							
4. Marital conflict	1.83	0.85	0.06	0.04	0.04	-						
5. Open expression	3.69	0.90	−0.11 *	0.02	0.05	−0.40 **	-					
6. Listening to parents	3.96	0.78	−0.09	0.04	0.08	−0.42 **	0.68 **	-				
7. Conflict resolution	3.71	0.96	−0.06	0.03	0.04	−0.49 **	0.72 **	0.75 **	-			
8. Mutual understanding	3.82	0.77	−0.08	0.03	0.05	−0.40 **	0.67 **	0.74 **	0.75 **	-		
9. Peer attachment	3.52	0.75	0.04	−0.01	0.18 **	−0.28 **	0.47 **	0.46 **	0.48 **	0.47 **	-	
10. Depressive symptoms	0.75	0.50	0.17 **	0.01	0.00	0.35 **	−0.43 **	−0.42 **	−0.40 **	−0.42 **	−0.48 **	-

Note. Gender: 1 = male; 2 = female; * *p* < 0.05, ** *p* < 0.01.

## Data Availability

Due to ethical restrictions, some access restrictions apply to the data underlying the findings. However, reasonable requests to access the datasets should be directed to the corresponding author after obtaining the permission from Zhejiang Sci-Tech University, China and signing the usage agreement for research purpose.
